# Positive association between weight-adjusted-waist index and hyperuricemia in patients with hypertension: The China H-type hypertension registry study

**DOI:** 10.3389/fendo.2022.1007557

**Published:** 2022-10-06

**Authors:** Peixu Zhao, Weidong Shi, Yumeng Shi, Yurong Xiong, Congcong Ding, Xiaoli Song, Guosheng Qiu, Junpei Li, Wei Zhou, Chao Yu, Tao Wang, Lingjuan Zhu, Xiaoshu Cheng, Huihui Bao

**Affiliations:** ^1^ Department of Cardiovascular Medicine, The Second Affiliated Hospital of Nanchang University, Nanchang, China; ^2^ Jiangxi Provincial Cardiovascular Disease Clinical Medical Research Center, Nanchang, China; ^3^ Jiangxi Sub-Center of National Clinical Research Center for Cardiovascular Diseases, Nanchang, China; ^4^ Department of Cardiology, Wuyuan People’s Hospital, Wuyuan, China; ^5^ Center for Prevention and Treatment of Cardiovascular Diseases, The Second Affiliated Hospital of Nanchang University, Nanchang, China

**Keywords:** weight-adjusted-waist index, hyperuricemia, hypertension, obesity, Chinese population

## Abstract

**Background and aims:**

The relationship between the new obesity index weight-adjusted-waist index (WWI) and hyperuricemia is unclear. We aimed to explore the association of the WWI and hyperuricemia among the hypertensive population.

**Methods:**

A total of 14,078 hypertension participants with complete data were included in our study. WWI was calculated by waist circumference divided by the square root of weight. Specifically, men with 420 μmol/L and women with 360 μmol/L were considered to have hyperuricemia.

**Results:**

The prevalence of hyperuricemia was 61.1% in men and 51.4% in women. On the whole, multivariate logistic regression analyses found that there was a linear positive correlation of WWI with hyperuricemia in both men (OR: 1.37; 95%CI: 1.25, 1.49) and women (OR: 1.35; 95%CI: 1.26, 1.45). Subgroup analysis found that the relationship between WWI and hyperuricemia was stable in stratified subgroups (all *P*-interactions >.05).

**Conclusion:**

WWI showed a positive association with hyperuricemia among hypertension patients.

## Introduction

The incidence rate and prevalence of hyperuricemia are on the rise worldwide. Based on the results of two nationally representative cross-sectional surveys conducted in China, the prevalence of hyperuricemia among Chinese adults has risen from 11.1% in 2015–2016 to 14.0% in 2018–2019 (1). Survey data in America indicates that hyperuricemia affected about 43.3 million people and was prevalent in 21.4% of the population (2). In Japan, approximately 25.8% suffered from hyperuricemia (3). Hyperuricemia can cause kidney stones and gout (4). In addition, high serum uric acid (SUA) is also closely related to hypertension (5), cardiovascular disease (6), chronic kidney disease (7), diabetes (8), and obesity (9). The long-term prevention and treatment of hyperuricemia causes a heavy burden on economic development and people’s lives and health worldwide. Therefore, we urgently need to find a changeable and measurable index to reduce the occurrence of hyperuricemia risk events.

Uric acid is the final metabolite of human purine compounds and is mainly excreted by the kidney. Hyperuricemia results from the body producing too much uric acid or excreting it insufficiently (10). Relevant studies show that excessive fat deposition in obese patients acts on the liver and adds to the production of uric acid (11). In addition, obesity can also cause insulin resistance, raise the risk of kidney damage, and impair uric acid elimination from the kidney (12, 13). It is worth noting that a variety of cytokines secreted by adipocytes also promote the production of uric acid through the regulation of the human metabolism (14). Many epidemiological studies have found that body mass index (BMI), which is the commonly used obesity index, has been related to SUA and hyperuricemia for a long time (15–17). Recently, obesity based on BMI has been questioned. BMI cannot distinguish between fat and muscle mass, so its association with hyperuricemia is not stable. A cross-sectional study conducted by Huang et al. in 1284 members of the Chinese general population found that there was no significant relationship between BMI and hyperuricemia (18). Accordingly, increasing studies have explored the connection between new obesity indicators, SUA, and hyperuricemia. The weight-adjusted-waist index (WWI) is a new obesity anthropometric index proposed in 2018 (19). Compared with BMI, WWI can better distinguish fat and muscle mass components, and it mainly reflects the problem of central obesity and is not affected by weight (20).

As we all know, obesity is a risk element for hypertension (21). Previous epidemiological studies also show that hyperuricemia is an independent risk factor for hypertension (22). Relevant studies confirm, that WWI can predict the occurrence of hypertension and is better than BMI (23). However, there is no study to explore the effect of WWI on SUA level and hyperuricemia in hypertensive patients. Considering the higher risk of adverse events in this high-risk group, it is essential to make clear the exact association between WWI, SUA level, and hyperuricemia in patients with hypertension. Therefore, this study aims to assess the association between WWI, SUA level, and hyperuricemia risk in the Chinese H-type hypertension population and clarify the dose-response relationship between them to provide the basis for early identification of patients with hyperuricemia.

## Methods

### Participants

The data analyzed in this study was derived from the China Hypertension Registry Study (Registration number: ChiCTR1800017274). The research design and methods have been published in previous articles (24). Briefly, the study was conducted in Wuyuan County in Jiangxi Province of China. Patients with hypertension and aged ≥18 years were eligible participants. Any one of sitting systolic blood pressure (SBP) ≥140 mmHg, diastolic blood pressure (DBP) ≥90 mmHg, or taking antihypertensive agents was regarded as hypertension. The exclusion criteria of this study included (1) inability to prove informed consent due to psychological or nervous system injury, (2) not able to follow up according to the protocol or due to a planned relocation, and (3) study physicians deem patient not suitable for inclusion or unable to complete follow-up. The ethics committee of the Institute of Biomedical Research of Anhui Medical University approved the protocol. All participants signed informed consent.

A total of 14,268 participants completed the survey. We excluded participants who were nonhypertensive (*n* = 34) and had data missing (*n* = 156). Finally, 14,078 participants were enrolled in our analysis (**Figure S1**).

### Data collection

Demographic information, including sex, age, lifestyle data (smoking and drinking), medication information, and medical history, was gathered by questionnaire used by the research staff. After having a rest for 10 minutes, an electronic sphygmomanometer (Omron; Dalian, China) was used to acquire the participants’ information on blood pressure; the measurement was repeated three times with an interval of 1 minute. The mean value of three independent measurements for SBP and DBP were taken for analysis. Blood samples were obtained by venipuncture at baseline and processed and analyzed at Biaojia Biotechnology, sited in Shenzhen in Guangdong Province, China, for homocysteine, serum total cholesterol, triglyceride, uric acid, high density lipoprotein, low density lipoprotein, and eGFR.

### Anthropometric measurements

Anthropometric indices, such as height, weight, and waist circumference (WC), were also collected. The height was obtained by using a fixed vertical ruler and standard right angle device on participants without shoes. Weight was also gained without shoes. WC was measured when standing with a tape measure at the end of expiration. BMI was calculated by dividing body weight (kg) by the square of height (m). WWI was calculated as WC (cm) divided by the square root of weight (kg) (19).

Based on previous studies on obesity and hyperuricemia, we divided WWI into four groups according to its quartile. WWI quartiles in males were defined as follows: <10.4 cm/√kg (Q 1), ≥10.4 and <10.8cm/√kg (Q 2), ≥10.8 and <11.2 cm/√kg (Q 3), and ≥11.2 cm/√kg (Q 4). WWI quartiles in females were <10.8 cm/√kg (Q 1), ≥10.8 and <11.3cm/√kg (Q 2), ≥11.3 and <11.8 cm/√kg (Q 3), and ≥11.8 cm/√kg (Q 4).

### Definition of hyperuricemia

The level of SUA in the present study was measured by automated clinical analyzers (Beckman Coulter). Specifically, men with 420 μmol/L and women with 360 μmol/L were considered to have hyperuricemia (25, 26).

### Statistical analysis

Baseline characteristics described continuous variables with mean ± SD and categorical variables with percentage (%). The Chi-square (x2) tests or ANOVA were used to evaluate the differences among the groups by WWI quartiles. Multivariate logistic regression was used to evaluate the OR and 95% CI of the correlation between WWI and hyperuricemia. The fitted smoothing curve and generalized additive model were also used to examine the dose-response correlation of WWI with SUA and the risk of hyperuricemia. At the same time, we converted WWI into classification variables and calculated the *P* trend value. We constructed three models to evaluate the independent correlation between WWI with SUA and hyperuricemia. Model 1 was a crude model, unadjusted. Model 2 was adjusted for age, current smoking, and current drinking. Model 3 was adjusted for age, current smoking, current drinking, heart rate, stroke, Diabetes mellitus, coronary heart disease, antihypertensive drugs, lipid-lowering drugs, glucose-lowering drugs, homocysteine, serum total cholesterol, triglyceride, high density lipoprotein, low density lipoprotein, and eGFR. All the covariates were selected on the basis of their clinical importance, statistical significance in the univariable analyses, and the estimated variables change of at least 10% of potential confounding effects (27). Moreover, we also performed subgroup analyses to test the robustness of the results in different subgroups.

The statistical package R (R Foundation for statistical Computing) and Empower (R) (www.empowerstats.com) were used to perform all analyses. A two-tailed *P* <.05 was deemed statistically significant.

## Results

### Baseline characteristics of study participants

The quartiles of the WWI show participants’ baseline characteristics in **Table 1**: 6695 men and 7383 women with hypertension with complete data were brought into the final analysis. The mean age of men was 63.8 ± 9.8 years and that of women was 63.9 ± 8.9 years. The mean WWI of men was 10.8 ± 0.7 cm/√kg and that of women was 11.4 ± 0.8 cm/√kg. The BMI, WC, coronary heart disease, glucose-lowering drugs, and uric acid were significantly higher in the higher WWI group in both men and women. In men, higher WWI levels were correlated with higher levels of antihypertensive drugs, triglycerides, low density lipoprotein, and lower levels of current drinkers and current smokers. In women, stroke and homocysteine were significantly higher and eGFR levels were lower in the group with higher WWI.

**Table 1:** Baseline characteristics of participants stratified by quartiles of weight-adjusted-waist index (WWI).

Variables	Total	WWI (cm/√kg)				P value
		Q1 (<10.4)	Q2 (≥10.4, <10.8)	Q3 (≥10.8, <11.2)	Q4 (≥11.2)	
Men						
n	6695	1674	1673	1674	1674	
Age, y	63.8 ± 9.8	63.5 ± 9.4	62.7 ± 9.8	63.3 ± 10.0	65.7 ± 9.7	<0.001
BMI, kg/m2	23.4 ± 3.9	21.4 ± 4.7	23.2 ± 3.2	24.1 ± 3.0	24.9 ± 3.5	<0.001
WC, cm	84.3 ± 9.9	75.0 ± 7.4	82.9 ± 7.4	87.1 ± 7.1	92.3 ± 8.5	<0.001
Heart rate, bpm	74.6 ± 13.9	73.0 ± 13.7	73.7 ± 13.5	74.8 ± 13.2	76.9 ± 14.9	<0.001
Stroke, n (%)	566 (8.5)	136 (8.1)	133 (7.9)	146 (8.7)	151 (9.0)	0.652
Diabetes mellitus, n (%)	1068 (16.0)	183 (10.9)	226 (13.5)	314 (18.8)	345 (20.6)	<0.001
Coronary heart disease, n (%)	368 (5.5)	74 (4.4)	78 (4.7)	93 (5.6)	123 (7.3)	<0.001
Current smoking, n (%)	3236 (48.3)	878 (52.5)	788 (47.1)	787 (47.0)	783 (46.8)	0.002
Current drinking, n (%)	2668 (39.9)	693 (41.4)	682 (40.8)	666 (39.8)	627 (37.5)	0.096
Medication use, n (%)						
Antihypertensive drugs	4324 (64.6)	1055 (63.1)	1068 (63.8)	1081 (64.6)	1120 (66.9)	0.109
Lipid-lowering drugs	235 (3.5)	50 (3.0)	53 (3.2)	73 (4.4)	59 (3.5)	0.138
Glucose-lowering drugs	287 (4.3)	47 (2.8)	58 (3.5)	83 (5.0)	99 (5.9)	<0.001
Laboratory results, mean						
Homocysteine, μmol/L	20.6 ± 13.8	20.5 ± 13.3	20.2 ± 13.3	20.6 ± 14.7	20.9 ± 13.9	0.573
Serum total cholesterol, mmol/L	4.9 ± 1.1	4.9 ± 1.0	4.9 ± 1.0	5.0 ± 1.1	5.0 ± 1.1	0.006
Triglyceride, mmol/L	1.7 ± 1.3	1.3 ± 1.1	1.7 ± 1.2	1.8 ± 1.4	1.9 ± 1.2	<0.001
Uric acid, mmol/L	465.6 ± 118.7	443.3 ± 113.2	459.5 ± 114.0	474.0 ± 119.7	485.7 ± 123.5	<0.001
High density lipoprotein, mmol/L	1.5 ± 0.4	1.7 ± 0.5	1.5 ± 0.4	1.5 ± 0.4	1.4 ± 0.4	<0.001
Low density lipoprotein, mmol/L	2.8 ± 0.8	2.7 ± 0.7	2.8 ± 0.8	2.9 ± 0.8	3.0 ± 0.8	<0.001
eGFR, mL/min/1.73 m^2^	85.7 ± 20.4	86.6 ± 20.7	86.8 ± 19.9	86.1 ± 20.6	83.3 ± 20.2	<0.001
		Q1 (<10.8)	Q2 (≥10.8, <11.3)	Q3 (≥11.3, <11.8)	Q4 (≥11.8)	*P value*
Women,						
n	7383	1846	1845	1846	1846	
Age, y	63.9 ± 8.9	61.0 ± 9.3	62.9 ± 8.4	64.3 ± 8.3	67.3 ± 8.5	<0.001
BMI, kg/m2	23.8 ± 3.6	22.1 ± 3.5	23.5 ± 3.2	24.5 ± 3.4	25.1 ± 3.5	<0.001
WC, cm	83.4 ± 9.8	74.3 ± 7.4	81.7 ± 6.8	86.1 ± 7.3	91.4 ± 8.5	<0.001
Heart rate, bpm	78.6 ± 14.2	78.1 ± 15.3	78.1 ± 13.5	78.3 ± 13.9	79.9 ± 13.8	<0.001
Stroke, n (%)	409 (5.5)	93 (5.0)	97 (5.3)	107 (5.8)	112 (6.1)	0.497
Diabetes mellitus, n (%)	1520 (20.6)	262 (14.2)	373 (20.2)	417 (22.6)	468 (25.4)	<0.001
Coronary heart disease, n (%)	360 (4.9)	82 (4.4)	83 (4.5)	84 (4.6)	111 (6.0)	0.076
Current smoking, n (%)	406 (5.5)	88 (4.8)	95 (5.2)	87 (4.7)	136 (7.4)	<0.001
Current drinking, n (%)	380 (5.1)	93 (5.0)	95 (5.2)	104 (5.6)	88 (4.8)	0.687
Medication use, n (%)						
Antihypertensive drugs	4797 (65.0)	1121 (60.7)	1220 (66.2)	1218 (66.0)	1238 (67.1)	<0.001
Lipid-lowering drugs	266 (3.6)	55 (3.0)	77 (4.2)	67 (3.6)	67 (3.6)	0.284
Glucose-lowering drugs	456 (6.2)	68 (3.7)	108 (5.9)	132 (7.2)	148 (8.0)	<0.001
Laboratory results, mean						
Homocysteine, μmol/L	15.8 ± 7.3	15.5 ± 7.9	15.6 ± 6.9	15.7 ± 7.0	16.4 ± 7.4	<0.001
Serum total cholesterol, mmol/L	5.4 ± 1.1	5.3 ± 1.1	5.4 ± 1.2	5.4 ± 1.1	5.3 ± 1.2	<0.001
Triglyceride, mmol/L	1.9 ± 1.3	1.6 ± 1.1	2.0 ± 1.3	2.0 ± 1.3	2.1 ± 1.3	<0.001
Uric acid, mmol/L	379.0 ± 106.0	355.3 ± 99.7	372.7 ± 103.9	383.6 ± 104.3	404.3 ± 110.1	<0.001
High density lipoprotein, mmol/L	1.6 ± 0.4	1.7 ± 0.4	1.6 ± 0.4	1.6 ± 0.4	1.5 ± 0.4	<0.001
Low density lipoprotein, mmol/L	3.1 ± 0.8	3.0 ± 0.8	3.2 ± 0.8	3.2 ± 0.8	3.2 ± 0.8	<0.001
eGFR, mL/min/1.73 m^2^	89.8 ± 19.6	92.6 ± 19.1	91.1 ± 19.1	90.1 ± 18.8	85.5 ± 20.5	<0.001

BMI, Body mass index; WC, waist circumference; eGFR, estimated glomerular filtration rate.

### Association between WWI and hyperuricemia

**Table 2** describes the results of multivariate regression for correlation analyses of WWI with hyperuricemia. Generally, there were significant positive associations of WWI and hyperuricemia in both men and women. After fully adjusting for confounding factors, per one unit increase in WWI, the risk of hyperuricemia was raised by 37% (OR: 1.37; 95% CI: 1.25, 1.49) among men and by 35% (OR: 1.35; 95% CI: 1.26, 1.45) among women. Then, we grouped WWI as the categorical variable for further analysis. Among men, in model 3, compared with Q1, the risk of hyperuricemia was found raised in Q2 (OR: 1.25; 95% CI: 1.07, 1.45), Q3 (OR: 1.52; 95% CI: 1.30, 1.77), and Q4 (OR: 1.77; 95% CI: 1.50, 2.08), respectively (*P* for trend <.001). Among women, also taking the lowest quartile (Q1) as the reference group, the incidence of hyperuricemia raised with the increase of WWI, Q2 (OR: 1.20; 95% CI: 1.03, 1.39), Q3 (OR: 1.51; 95%CI: 1.30, 1.75), and Q4 (OR: 1.98; 95% CI 1.69, 2.31) respectively (*P* for trend <.001). The positive correlation between WWI with SUA both in men and women are shown in **Table S1**.

**Table 2:** Association between the weight-adjusted-waist index and hyperuricemia in different models.

WWI (cm/√kg)	N	Events, n (%)	Model 1		Model 2		Model 3	
			OR	95% CI	OR	95% CI	OR	95% CI
Men								
Per 1unit increment	6695	4090 (61.1)	1.42	1.32, 1.54	1.43	1.33, 1.55	1.37	1.25, 1.49
Q1 (<10.4)	1674	880 (52.6)	1		1		1	
Q2 (≥10.3, <10.8)	1673	1002 (59.9)	1.35	1.17, 1.55	1.33	1.16, 1.53	1.25	1.07, 1.45
Q3 (≥10.8, <11.2)	1674	1072 (64.0)	1.61	1.40, 1.85	1.60	1.39, 1.84	1.52	1.30, 1.77
Q4 (≥11.2)	1674	1136 (67.9)	1.91	1.66, 2.19	1.93	1.68, 2.23	1.77	1.50, 2.08
P for trend			<0.001		<0.001		<0.001	
Women								
Per 1unit increment	7383	3797 (51.4)	1.45	1.37, 1.55	1.41	1.32, 1.50	1.35	1.26, 1.45
Q1 (<10.8)	1846	762 (41.3)	1		1		1	
Q2 (≥10.8, <11.3)	1845	895 (48.5)	1.34	1.18, 1.53	1.31	1.15, 1.49	1.20	1.03, 1.39
Q3 (≥11.3, <11.8)	1846	994 (53.8)	1.66	1.46, 1.89	1.60	1.40, 1.82	1.51	1.30, 1.75
Q4 (≥11.8)	1846	1146 (62.1)	2.33	2.04, 2.66	2.18	1.90, 2.50	1.98	1.69, 2.31
P for trend			<0.001		<0.001		<0.001	

Model 1 was adjusted for none.

Model 2 was adjusted for Age, Current smoking, Current drinking.

Model 3 was adjusted for Age, Current smoking, Current drinking, Heart rate, Stroke, Diabetes mellitus, Coronary heart disease, Antihypertensive drugs, Lipid-lowering drugs, Glucose-lowering drugs, Homocysteine, Serum total cholesterol, Triglyceride, High density lipoprotein, Low density lipoprotein, eGFR.

**Figure 1:**
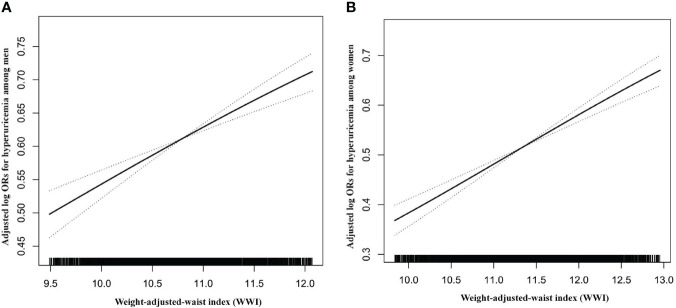
Dose–response relationship between WWI and hyperuricemia. **(A)** Men; **(B)** Women. All adjusted for age, heart rate, stroke, diabetes mellitus, coronary heart disease, current smoking, current drinking, antihypertensive drugs, lipid-lowering drugs, glucose-lowering drugs, homocysteine, serum total cholesterol, triglyceride, high density lipoprotein, low density lipoprotein, and eGFR.

We also used the fitted smoothing curve and generalized additive model to confirm the linearly positive association between WWI with SUA and hyperuricemia for men and women (**Figure 1**, **Figure S2**).

### Sensitivity analyses

We also conducted a sensitivity analysis to assess whether BMI has a confounding effect on the association of WWI and the risk of hyperuricemia. As shown in **Table S2**, the correlation of WWI (continuous and categorical variables) with hyperuricemia was not changed by adjusting BMI. Even when BMI was well-controlled (normal BMI: ≥18.5, <24 Kg/m2), the correlation of WWI and hyperuricemia remain significant (**Table S3**).

### Subgroup analyses

**Figure 2:**
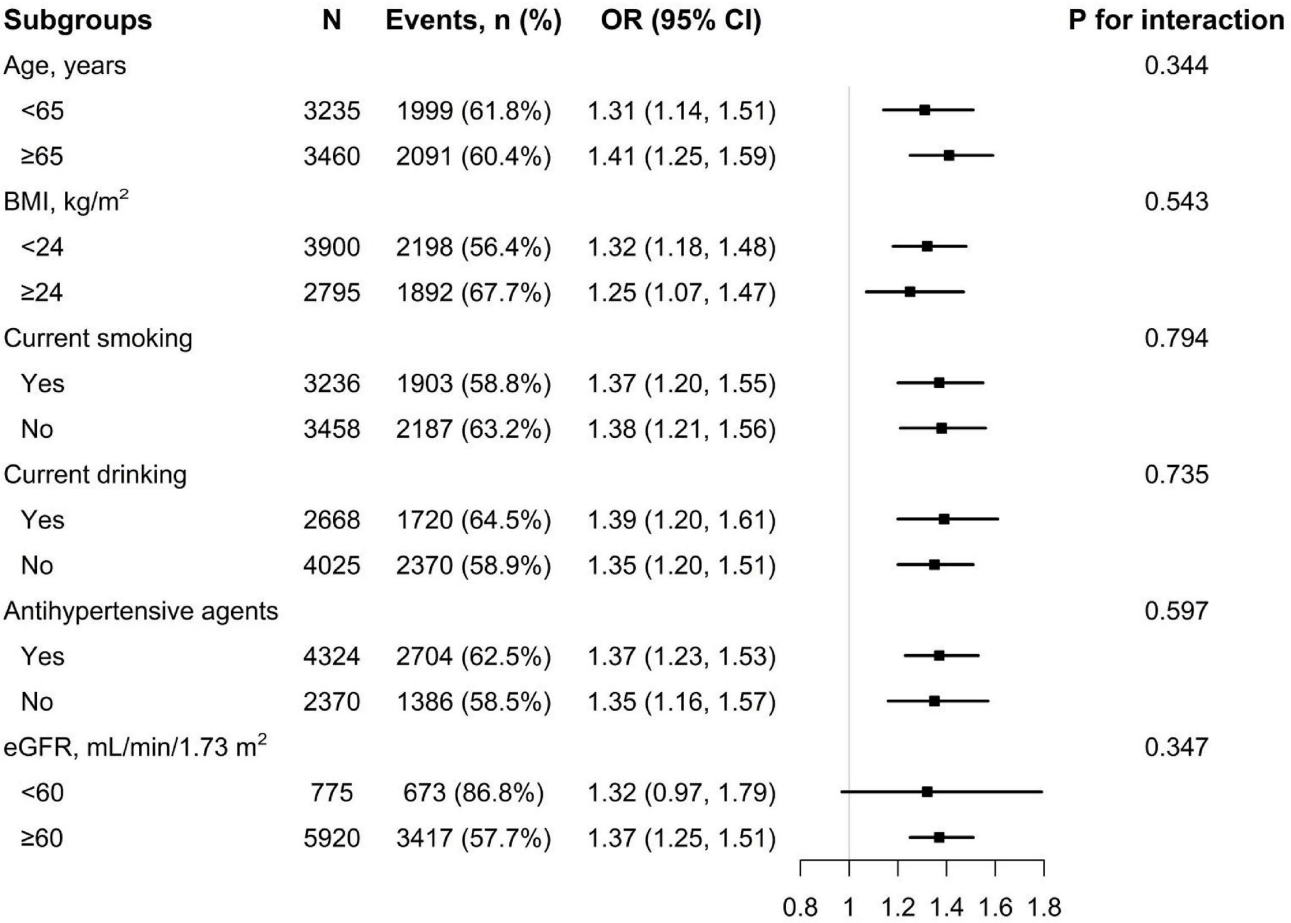
Subgroup analysis of the association between WWI and hyperuricemia among men. Each subgroup analysis is adjusted if not stratified for age, heart rate, stroke, diabetes mellitus, coronary heart disease, current smoking, current drinking, antihypertensive drugs, lipid-lowering drugs, glucose-lowering drugs, homocysteine, serum total cholesterol, triglyceride, high density lipoprotein, low density lipoprotein, and eGFR.

**Figure 3:**
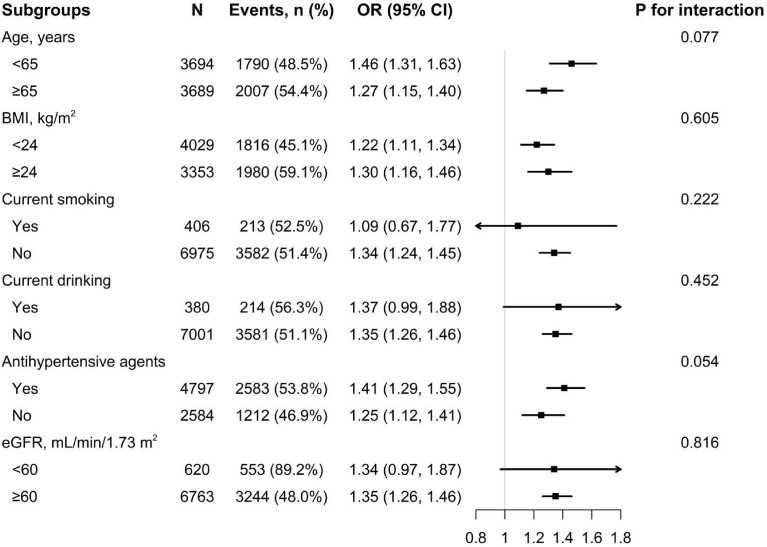
Subgroup analysis of the association between WWI and hyperuricemia among women. Each subgroup analysis is adjusted if not stratified for age, heart rate, stroke, diabetes mellitus, coronary heart disease, current smoking, current drinking, antihypertensive drugs, lipid-lowering drugs, glucose-lowering drugs, homocysteine, serum total cholesterol, triglyceride, high density lipoprotein, low density lipoprotein, and eGFR.

To confirm whether the correlation of WWI with hyperuricemia is stable in different subgroups, subgroup analyses were performed (**Figures 2**, **3**). The results show that no significant differences were found in the subgroups of age, BMI, current smoking, current drinking, antihypertensive agents, and eGFR among both men and women (all *P* for interaction >.05).

## Discussion

In this cross-sectional study based on a large sample size, we examined the correlation of WWI, a new obesity index, with hyperuricemia in people with hypertension for the first time. In the fully adjusted model, we found a positive correlation of WWI with SUA level and the risk of hyperuricemia in both men and women, and these results were stable in subgroup analyses. Even after adjusting for BMI and when BMI was well-controlled (normal BMI: ≥18.5, <24 Kg/m2), the results remain significant.

Previous studies report that the traditional obesity index, BMI, is related to SUA and hyperuricemia changes. A large cross-sectional study included 90,047 Japanese and 14,734 American participants, and the results showed that higher BMI was an independent risk element for hyperuricemia in both Japanese and American populations (15). Previously, Ishizaka et al. conducted a 2-year routine health screening for 3153 participants and analyzed their data, demonstrating that there was a positive correlation of BMI with SUA concentration (16). Similarly, through the retrospective analysis of 39,736 healthy subjects, Wang et al. reported that BMI was positively correlated with SUA (17). Compared with those who were underweight (BMI < 18.5kg/m^2^), the prevalence of hyperuricemia in overweight people (BMI: 23–27.5kg/m^2^) was about 2.98 times more and that in obese people (BMI ≥ 27.5kg/m^2^) was 5.96 times more. However, another study, also aimed at the Chinese population, indicated that there was a short-of-significant correlation of BMI with hyperuricemia (18). This phenomenon may be due to the limitation of BMI (28) and the existence of the obesity paradox (16, 29).

In order to better explore the correlation between obesity and hyperuricemia, some recent studies tend to use nontraditional obesity indicators as a measure of obesity to examine the exact relationship between them. A cross-sectional study of 174,698 Chinese adults by Liu et al. aimed to explore the correlation between new obesity indicators and hyperuricemia. The results show that, after adjusting for confounding factors, the cardiometabolic index (CMI) and lipid accumulation product (LAP) index highest quartile groups’ OR were 2.049 (95%CI: 1.824, 2.302; *p* <.001) and 4.332 (95%CI:3.938, 4.765; *p* <.001), respectively (30). Another cross-sectional study of 1284 ordinary people by Huang et al. showed that, after dividing the fatty liver index (FLI) and visceral adiposity index (VAI) into three groups, the risk of hyperuricemia in the highest third was 3.58 and 3.11 times that in the lowest third (18). Previously, a cross-sectional study of 11,345 participants found that both the body roundness index (BRI) and body shape index (ABSI) were significantly associated with hyperuricemia (31). However, most of these nontraditional obesity indicators are complex to calculate and have poor operability in practical application, and they were discussed in the general population.

WWI is a new obesity anthropometric index (19) proposed by Park et al. in 2018. It is calculated as WC (cm) divided by the square root of weight (kg), and under this background, WWI may weaken the correlation with BMI so as to mainly reflect the true central obesity independent of body weight. In a cross-sectional study of 602 65-year-old participants in the Anshan geriatric study, Kim et al, found that WWI can better distinguish fat and muscle mass components compared with BMI (20). Similarly, the prospective cohort study conducted by Ding et al. in 12,447 hypertensive participants also confirmed that WWI can better identify obesity than BMI to a certain extent (32). It is well-known that hypertension is a high-risk element for hyperuricemia (33). Li et al. conducted a cohort study containing 10,338 nonhypertensive participants and found that, compared with the lowest group of the four WWI groups, the risk ratio of hypertensive events in the highest group was 1.50 (95% CI: 1.24, 1.82; *P* <.001); that is, WWI was significantly correlated with the risk of hypertension (23). However, data on the impact of WWI on the high-risk population with high SUA hypertension is still lacking. In our study, we first report a positive correlation between WWI and hyperuricemia in patients with hypertension, and this relationship was stable even after adjusting for BMI and when BMI was well-controlled (normal BMI: ≥18.5, <24 Kg/m^2^).

The potential mechanism of the positive correlation between WWI and hyperuricemia can be explained by the role of abdominal fat as a marker of ectopic fat excess. The increase of WWI may reflect the dysfunction of adipose tissue, thereby causing an increase in uric acid secretion and inhibiting uric acid excretion. First, excessive fat deposition in obese patients will act on the liver, affect the metabolism of purine (34), and raise the production of uric acid (11). Second, obesity can cause insulin resistance, raise the risk of renal damage, and then damage the renal treatment of uric acid (12, 13). It is worth noting that a variety of cytokines secreted by adipocytes (such as adipokines and leptin) also promote the production of uric acid through the regulation of human metabolism (14, 35, 36).

The main advantages of our study were the large population-based sample size, including a large number of patients with hypertension, and subgroup analysis to test the robustness of the results. However, this study also has some limitations that need attention. First, this study was a cross-sectional design, so we were unable to determine the causal relationship correlation of WWI with hyperuricemia. Second, although we adjusted for possible covariates, the potential residual confounding factors may still exist. Third, recent epidemiological studies show that dietary factors are also a cause of hyperuricemia (37); however, our study did not collect the dietary status of uric acid metabolism, such as seafood and animal offal. Fourth, information on the use of drugs to reduce uric acid was not collected and may affect the diagnosis of hyperuricemia. However, considering that other studies did not include this factor, we believe that our results are still reliable. Fifth, the participants in this study are mainly concentrated in southern China. Therefore, whether these conclusions can be extrapolated to other nationalities remains to be further studied.

## Conclusion

In the hypertensive population, we found an independent positive relationship of WWI and the occurrence of hyperuricemia risk events. The results suggest that the WWI index can be used as a simple and effective intervention indicator and may have preventive value for the hyperuricemia population in southern China.

## Data availability statement

The original contributions presented in the study are included in the article/**Supplementary Material**. Further inquiries can be directed to the corresponding authors.

## Ethics statement

All procedures performed in studies involving human participants were in accordance with the ethical standards of the institutional and/or national research committee and with the 1964 Helsinki declaration and its later amendments or comparable ethical standards. The protocol was approved by the ethics committee of the Institute of biomedical research of Anhui Medical University. All participants provided written informed consent before entering the study.

## Author contributions

PZ wrote the manuscript. PZ, YX, and YS participated in the literature search, data analysis, and data interpretation. WS extracted and collected data. XS, GQ, CD, JL, WZ, CY, TW, and LZ conceived of the study and participated in its design and coordination. XC and HB participated in the study design and provided critical revision. All authors read and approved the final manuscript.

## Funding

This work was supported by the Jiangxi Science and Technology Innovation Platform Project (20165BCD41005), Jiangxi Provincial Natural Science Foundation (20212ACB206019), Jiangxi Science and Technology Innovation Base Construction Project (20221ZDG02010), Jiangxi Provincial Health Commission Science and Technology Project (202210495), Fund project of the Second Affiliated Hospital of Nanchang University (2016YNQN12034, 2019YNLZ12010, 2021efyA01, 2021YNFY2024).

## Acknowledgments

Thanks to all the investigators and subjects who participated in the China Hypertension Registry Study.

## Conflict of interest

The authors declare that the research was conducted in the absence of any commercial or financial relationships that could be construed as a potential conflict of interest.

## Publisher’s note

All claims expressed in this article are solely those of the authors and do not necessarily represent those of their affiliated organizations, or those of the publisher, the editors and the reviewers. Any product that may be evaluated in this article, or claim that may be made by its manufacturer, is not guaranteed or endorsed by the publisher.
