# Supplementary material for: Positive association between weight-adjusted-waist index and hyperuricemia in patients with hypertension: The China H-type hypertension registry study
Source: Front Endocrinol (Lausanne). 2022 Oct 6;13:1007557. doi: 10.3389/fendo.2022.1007557 (PMC9582276; doi:10.3389/fendo.2022.1007557)
Supplement: Supplementary file 1 [file DataSheet_1.docx]

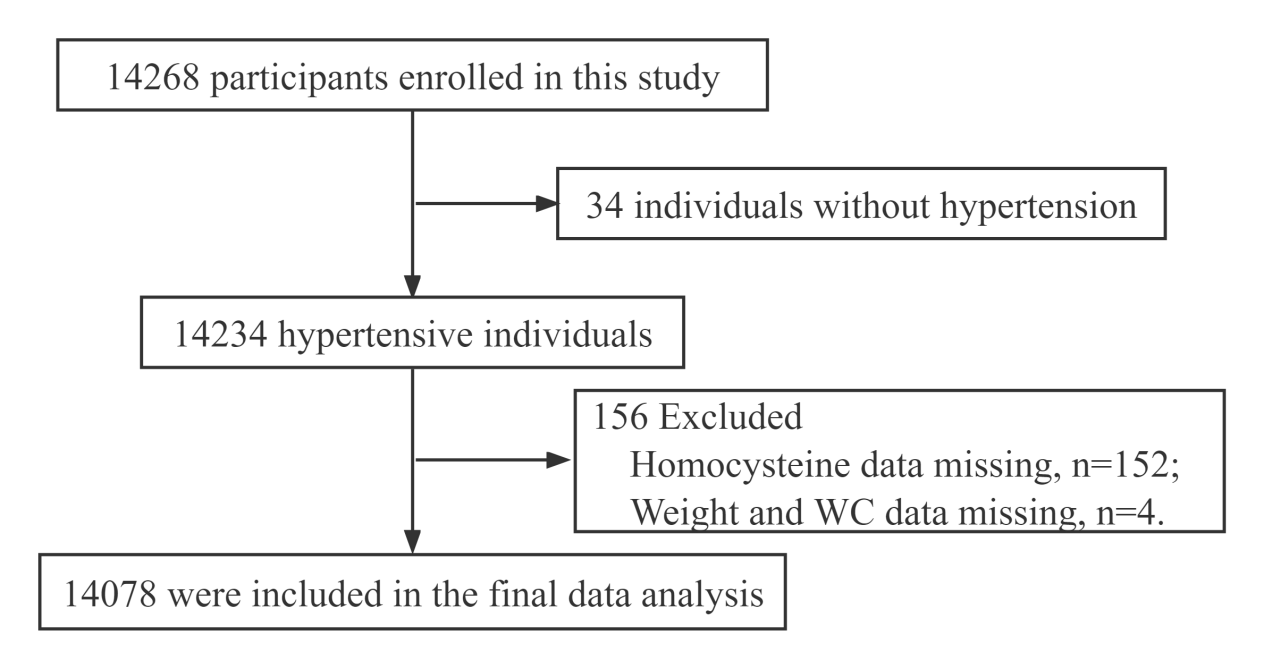


**Figure S1. Flow chart of participants.**

**Table S1. Association between the weight-adjusted-waist index and serum uric acid in different models.**

| WWI (cm/√kg) | Model 1 | | Model 2 | | Model 3 | |
| --- | --- | --- | --- | --- | --- | --- |
|  | β (95% CI) | P value | β (95% CI) | P value | β (95% CI) | P value |
| Men |  | | | | | |
| Per 1unit increment | 23.62 (19.45, 27.79) | <0.001 | 23.89 (19.71, 28.07) | <0.001 | 18.09 (14.29, 21.89) | <0.001 |
| Q1 (<10.8) | 0 |  | 0 |  | 0 |  |
| Q2 (≥10.8, <11.3) | 16.20 (8.23, 24.17) | <0.001 | 15.49 (7.53, 23.46) | <0.001 | 9.80 (2.87, 16.74) | 0.006 |
| Q3 (≥11.3, <11.8) | 30.68 (22.71, 38.65) | <0.001 | 30.19 (22.23, 38.14) | <0.001 | 22.37 (15.32, 29.43) | <0.001 |
| Q4 (≥11.8) | 42.46 (34.49, 50.43) | <0.001 | 42.97 (34.98, 50.95) | <0.001 | 31.75 (24.51, 38.98) | <0.001 |
| P for trend | <0.001 | | <0.001 | | <0.001 | |
| Women |  | | | | | |
| Per 1unit increment | 20.39 (17.47, 23.32) | <0.001 | 18.36 (15.32, 21.39) | <0.001 | 13.22 (10.53, 15.91) | <0.001 |
| Q1 (<10.4) | 0 |  | 0 |  | 0 |  |
| Q2 (≥10.3, <10.8) | 17.44 (10.69, 24.18) | <0.001 | 16.08 (9.32, 22.84) | <0.001 | 8.91 (3.03, 14.79) | 0.003 |
| Q3 (≥10.8, <11.2) | 28.28 (21.54, 35.03) | <0.001 | 25.95 (19.15, 32.74) | <0.001 | 18.47 (12.49, 24.44) | <0.001 |
| Q4 (≥11.2) | 48.95 (42.20, 55.69) | <0.001 | 44.73 (37.77, 51.69) | <0.001 | 31.31 (25.12, 37.50) | <0.001 |
| P for trend | <0.001 | | <0.001 | | <0.001 | |
| Model 1 was adjusted for none. | | | | | | |
| Model 2 was adjusted for Age, Current smoking, Current drinking. | | | | | | |
| Model 3 was adjusted for Age, Current smoking, Current drinking, Heart rate, Stroke, Diabetes mellitus, Coronary heart disease, Antihypertensive drugs, Lipid-lowering drugs, Glucose-lowering drugs, Homocysteine, Serum total cholesterol, Triglyceride, High density lipoprotein, Low density lipoprotein, eGFR. | | | | | | |


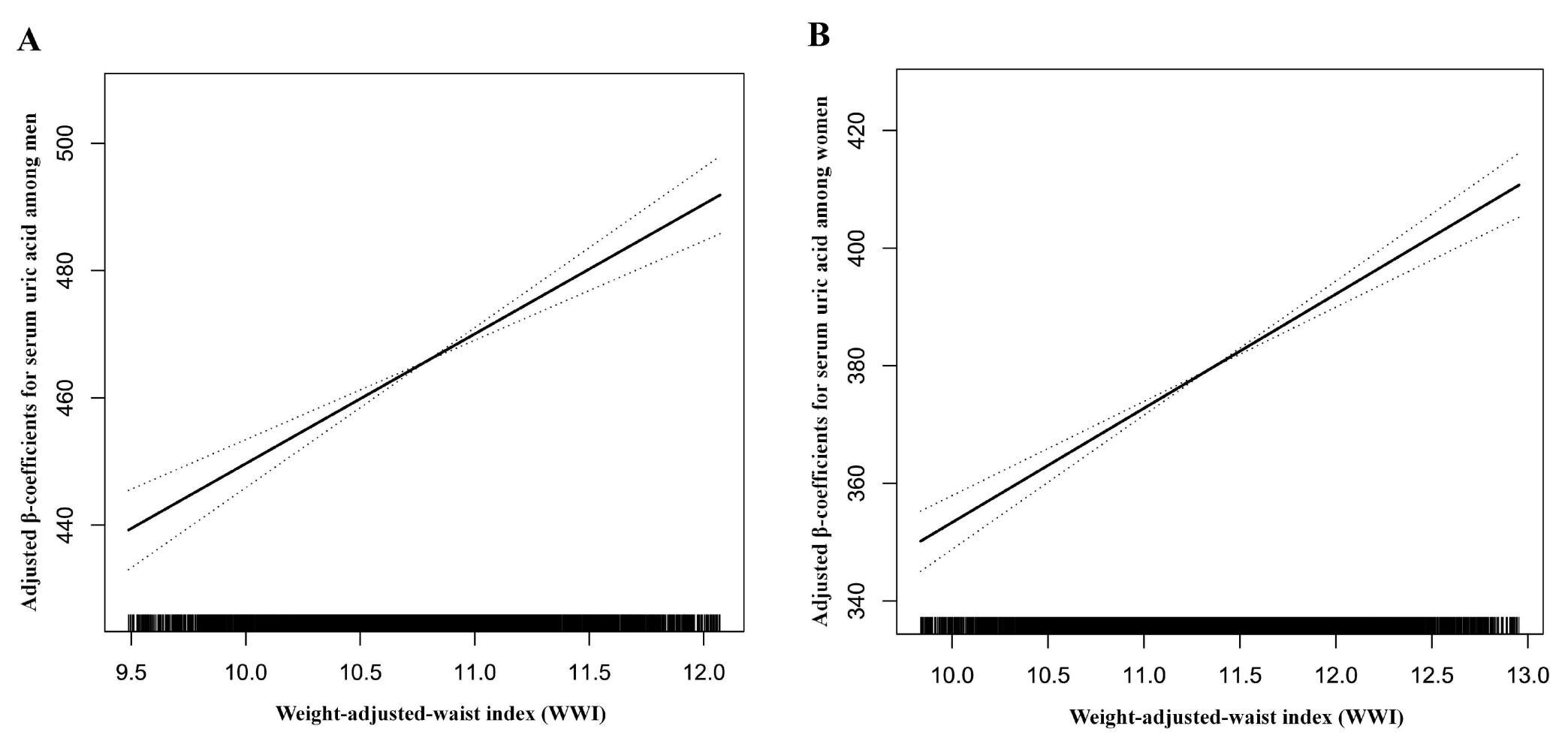


**Figure S2. Dose–response relationship between weight-adjusted-waist index and serum uric acid.** A. Men; B. Women. All adjusted for Age, Heart rate, Stroke, Diabetes mellitus, Coronary heart disease, Current smoking, Current drinking, Antihypertensive drugs, Lipid-lowering drugs, Glucose-lowering drugs, Homocysteine, Serum total cholesterol, Triglyceride, High density lipoprotein, Low density lipoprotein, eGFR.

**Table S2. Association between the WWI and hyperuricemia further adjustment for BMI.**

| WWI (cm/√kg) | N | Events, n(%) | Model 1 | | Model 2 | |
| --- | --- | --- | --- | --- | --- | --- |
|  |  |  | OR (95% CI) | P value | OR (95% CI) | P value |
| Men |  | | | | | |
| Per 1unit increment | 6695 | 4090(61.1) | 1.37 (1.25, 1.49) | <0.001 | 1.30 (1.18, 1.42) | <0.001 |
| Q1 (<10.4) | 1674 | 880(52.6) | 1 |  | 1 |  |
| Q2 (≥10.3, <10.8) | 1673 | 1002(59.9) | 1.25 (1.07, 1.45) | 0.005 | 1.20 (1.03, 1.40) | 0.023 |
| Q3 (≥10.8, <11.2) | 1674 | 1072(64.0) | 1.52 (1.30, 1.77) | <0.001 | 1.42 (1.21, 1.68) | <0.001 |
| Q4 (≥11.2) | 1674 | 1136(67.9) | 1.77 (1.50, 2.08) | <0.001 | 1.61 (1.35, 1.92) | <0.001 |
| P for trend |  |  | <0.001 | | <0.001 | |
| Women |  | | | | | |
| Per 1unit increment | 7383 | 3797(51.4) | 1.35 (1.26, 1.45) | <0.001 | 1.22 (1.14, 1.32) | <0.001 |
| Q1 (<10.8) | 1846 | 762(41.3) | 1 |  | 1 |  |
| Q2 (≥10.8, <11.3) | 1845 | 895(48.5) | 1.20 (1.03, 1.39) | 0.017 | 1.09 (0.94, 1.27) | 0.240 |
| Q3 (≥11.3, <11.8) | 1846 | 994(53.8) | 1.51 (1.30, 1.75) | <0.001 | 1.29 (1.11, 1.50) | 0.001 |
| Q4 (≥11.8) | 1846 | 1146(62.1) | 1.98 (1.69, 2.31) | <0.001 | 1.59 (1.35, 1.88) | <0.001 |
| P for trend |  |  | <0.001 | | <0.001 | |
| Model 1 was adjusted for Age, Current smoking, Current drinking, Heart rate, Stroke, Diabetes mellitus, Coronary heart disease, Antihypertensive drugs, Lipid-lowering drugs, Glucose-lowering drugs, Homocysteine, Serum total cholesterol, Triglyceride, High density lipoprotein, Low density lipoprotein, eGFR. | | | | | | |
| Model 2 was adjusted for Age, Current smoking, Current drinking, Heart rate, Stroke, Diabetes mellitus, Coronary heart disease, Antihypertensive drugs, Lipid-lowering drugs, Glucose-lowering drugs, Homocysteine, Serum total cholesterol, Triglyceride, High density lipoprotein, Low density lipoprotein, eGFR, BMI. | | | | | | |

**Table S3. Association between the weight-adjusted-waist index and hyperuricemia in individuals with different BMI groups.**

| WWI (cm/√kg) | N | Events, n(%) | Model 1 | | Model 2 | | Model 3 | |
| --- | --- | --- | --- | --- | --- | --- | --- | --- |
|  |  |  | OR (95% CI) | P value | OR (95% CI) | P value | OR (95% CI) | P value |
| BMI ＜18.5 Kg/m2 |  |  |  |  |  |  |  |  |
| Men |  | | | | | | | |
| Per 1unit increment | 490 | 267(54.5) | 1.14 (0.91, 1.43) | 0.238 | 1.11 (0.88, 1.40) | 0.378 | 1.04 (0.79, 1.38) | 0.753 |
| Q1 (<10.6) | 273 | 145(53.1) | 1 |  | 1 |  | 1 |  |
| Q2 (≥10.6, <11.1) | 105 | 58(55.2) | 1.09 (0.69, 1.71) | 0.711 | 1.05 (0.66, 1.66) | 0.847 | 0.88 (0.52, 1.48) | 0.622 |
| Q3 (≥11.1, <11.6) | 50 | 29(58) | 1.22 (0.66, 2.24) | 0.524 | 1.15 (0.62, 2.15) | 0.653 | 1.40 (0.69, 2.84) | 0.350 |
| Q4 (≥11.6) | 62 | 35(56.5) | 1.14 (0.66, 1.99) | 0.634 | 1.07 (0.61, 1.89) | 0.816 | 0.88 (0.45, 1.72) | 0.711 |
| P for trend |  |  | 0.497 | | 0.704 | | 0.980 | |
| Women |  |  |  | | | | | |
| Per 1unit increment | 430 | 180(41.9) | 0.85 (0.67, 1.08) | 0.177 | 0.77 (0.60, 0.99) | 0.042 | 0.80 (0.59, 1.08) | 0.148 |
| Q1 (<10.4) | 246 | 105(42.7) | 1 |  | 1 |  | 1 |  |
| Q2 (≥10.3, <10.8) | 93 | 40(43.0) | 1.01 (0.63, 1.64) | 0.957 | 0.90 (0.55, 1.47) | 0.669 | 0.73 (0.40, 1.34) | 0.308 |
| Q3 (≥10.8, <11.2) | 55 | 26(47.3) | 1.20 (0.67, 2.16) | 0.535 | 1.06 (0.58, 1.92) | 0.858 | 1.10 (0.56, 2.16) | 0.792 |
| Q4 (≥11.2) | 36 | 9(25) | 0.45 (0.20, 0.99) | 0.048 | 0.39 (0.17, 0.87) | 0.022 | 0.51 (0.20, 1.32) | 0.168 |
| P for trend |  |  | 0.265 | | 0.098 | | 0.352 | |
| BMI ≥18.5，＜24 Kg/m2 |  |  |  |  |  |  |  |  |
| Men |  | | | | | | | |
| Per 1unit increment | 3410 | 1931(56.6) | 1.36 (1.23, 1.52) | <0.001 | 1.36 (1.22, 1.52) | <0.001 | 1.39 (1.23, 1.58) | <0.001 |
| Q1 (<10.6) | 1106 | 548(49.5) | 1 |  | 1 |  | 1 |  |
| Q2 (≥10.6, <11.1) | 918 | 523(57.0) | 1.35 (1.13, 1.61) | <0.001 | 1.35 (1.13, 1.60) | 0.001 | 1.29 (1.06, 1.57) | 0.011 |
| Q3 (≥11.1, <11.6) | 776 | 478(61.6) | 1.63 (1.36, 1.97) | <0.001 | 1.63 (1.35, 1.96) | <0.001 | 1.59 (1.29, 1.97) | <0.001 |
| Q4 (≥11.6) | 610 | 382(62.6) | 1.71 (1.39, 2.09) | <0.001 | 1.70 (1.39, 2.10) | <0.001 | 1.75 (1.38, 2.21) | <0.001 |
| P for trend |  |  | <0.001 | | <0.001 | | <0.001 | |
| Women |  | | | | | | | |
| Per 1unit increment | 3599 | 1636(45.5) | 1.40 (1.29, 1.53) | <0.001 | 1.29 (1.18, 1.41) | <0.001 | 1.30 (1.17, 1.44) | <0.001 |
| Q1 (<10.4) | 1149 | 447(38.9) | 1 |  | 1 |  | 1 |  |
| Q2 (≥10.3, <10.8) | 970 | 415(42.8) | 1.17 (0.99, 1.40) | 0.070 | 1.11 (0.93, 1.32) | 0.251 | 1.01 (0.82, 1.23) | 0.956 |
| Q3 (≥10.8, <11.2) | 794 | 382(48.1) | 1.46 (1.21, 1.75) | <0.001 | 1.31 (1.09, 1.58) | 0.005 | 1.26 (1.02, 1.56) | 0.032 |
| Q4 (≥11.2) | 686 | 392(57.1) | 2.09 (1.73, 2.54) | <0.001 | 1.76 (1.44, 2.16) | <0.001 | 1.69 (1.34, 2.14) | <0.001 |
| P for trend |  |  | <0.001 | | <0.001 | | <0.001 | |
| BMI ≥24 Kg/m2 |  |  |  |  |  |  |  |  |
| Men |  | | | | | | | |
| Per 1unit increment | 2795 | 1892(67.7) | 1.28 (1.11, 1.47) | <0.001 | 1.30 (1.13, 1.51) | <0.001 | 1.25 (1.07, 1.47) | 0.006 |
| Q1 (<10.6) | 295 | 187(63.4) | 1 |  | 1 |  | 1 |  |
| Q2 (≥10.6, <11.1) | 650 | 421(64.8) | 1.06 (0.80, 1.41) | 0.682 | 1.05 (0.79, 1.40) | 0.730 | 1.08 (0.79, 1.47) | 0.646 |
| Q3 (≥11.1, <11.6) | 848 | 565(66.6) | 1.15 (0.87, 1.52) | 0.313 | 1.16 (0.88, 1.54) | 0.288 | 1.21 (0.89, 1.65) | 0.217 |
| Q4 (≥11.6) | 1002 | 719(71.8) | 1.47 (1.12, 1.93) | 0.006 | 1.51 (1.14, 2.01) | 0.004 | 1.48 (1.09, 2.03) | 0.013 |
| P for trend |  |  | 0.001 | | <0.001 | | 0.002 | |
| Women |  | | | | | | | |
| Per 1unit increment | 3353 | 1980(59.1) | 1.41 (1.28, 1.56) | <0.001 | 1.31 (1.18, 1.46) | <0.001 | 1.30 (1.16, 1.46) | <0.001 |
| Q1 (<10.4) | 451 | 210(46.6) | 1 |  | 1 |  | 1 |  |
| Q2 (≥10.3, <10.8) | 782 | 440(56.3) | 1.48 (1.17, 1.86) | 0.001 | 1.39 (1.10, 1.76) | 0.007 | 1.44 (1.11, 1.86) | 0.005 |
| Q3 (≥10.8, <11.2) | 997 | 586(58.8) | 1.64 (1.31, 2.05) | <0.001 | 1.49 (1.18, 1.87) | 0.001 | 1.60 (1.25, 2.06) | <0.001 |
| Q4 (≥11.2) | 1123 | 744(66.3) | 2.25 (1.80, 2.81) | <0.001 | 1.95 (1.54, 2.46) | <0.001 | 1.99 (1.54, 2.58) | <0.001 |
| P for trend |  |  | <0.001 | | <0.001 | | <0.001 | |
| Model 1 was adjusted for none. | | | | | | | | |
| Model 2 was adjusted for Age, Current smoking, Current drinking. | | | | | | | | |
| Model 3 was adjusted for Age, Current smoking, Current drinking, Heart rate, Stroke, Diabetes mellitus, Coronary heart disease, Antihypertensive drugs, Lipid-lowering drugs, Glucose-lowering drugs, Homocysteine, Serum total cholesterol, Triglyceride, High density lipoprotein, Low density lipoprotein, eGFR. | | | | | | | | |
